# Is it beneficial to use lateral protective crossing K-wires during medial open wedge high tibial osteotomy? A retrospective comparative study

**DOI:** 10.1186/s13018-025-05524-6

**Published:** 2025-02-06

**Authors:** Hossam Fathi Mahmoud, Ahmed Hatem Farhan, Ahmed Mohamed Abdelwahab, Ahmed Mohamed Elshaer, Mahmoud Abdo Mahmoud, Fahmy Samir Fahmy

**Affiliations:** 1 Orthopedic Surgery Department, Faculty of Medicine, Zagazig University, Sharkia, Egypt; 2 Orthopedic Surgery Department, Faculty of Medicine, Zagazig University, Sharkia, Egypt

**Keywords:** High tibial osteotomy, Lateral crossing K-wires, Lateral hinge fractures, KOOS-12, MPTA

## Abstract

**Background:**

An iatrogenic lateral hinge fracture is a common intraoperative problem that may occur during medial open wedge high tibial osteotomy (MOWHTO). This study aims to assess the significance of using additional crossing lateral K-wires and their advantage in protecting the lateral hinge during MOWHTO procedures.

**Methods:**

The data of patients fulfilling the inclusion criteria who underwent MOWHTO between May 2021 and August 2022 were retrospectively evaluated. One group had additional lateral hinge crossing K-wires (+ MOWHTO group), while the other did not (-MOWHTO group). Both groups were assessed for rate of intraoperative lateral hinge fractures, knee injury and osteoarthritis outcome score − 12 (KOOS-12), medial proximal tibial angle (MPTA), time of union, and time to return to work. The outcomes were compared using the independent T-test for continuous variables and the Fisher Exact test for nominal variables. A p-value of < 0.05 was considered statistically significant for both tests.

**Results:**

The study included forty-eight patients; twenty-four in each treatment group. The mean follow-up durations were 30.5 ± 3.6 months for + MOWHTO and 31.6 ± 3.2 months for –MOWHTO (*p* = 0.26). There was no statistically significant difference regarding mean age, sex, KOOS-12, MPTA, and time of surgery between both groups. The + MOWHTO group had a faster time of union (*p* = 0.001), an earlier return to work (*p* = 0.002), and a lower rate of intraoperative lateral hinge fractures (*p* = 0.04).

**Conclusion:**

This study demonstrated that using additional crossing lateral K-wires during MOWHTO had a beneficial effect on reducing the rate of iatrogenic lateral hinge fractures, with a faster time of union, and an early return to work. The KOOS-12, MPTA, and mean operative time did not reveal significant differences between treatment groups.

**Level of evidence:**

retrospective cohort comparative study; level III.

## Introduction

High tibial osteotomies (HTO) are joint-preserving surgeries proposed for the management of early joint arthritis in young adults with malalignment. The rationale of these surgeries is to correct the mechanical axis and shift the load from the arthritic compartment [1, 2]. Various surgical options exist, including opening and closing wedge osteotomies; however, the most common technique performed is the medial open wedge high tibial osteotomy (MOWHTO) [3, 4].

MOWHTO is primarily indicated for medial compartment degeneration with varus knee deformity. The goal of surgery is to delay the progression of joint degeneration by correcting the varus deformity and transferring the weight-bearing forces laterally [5–9]. Preservation of the bone stock, no need for fibular osteotomy, minimal risk of common peroneal nerve injuries, and early rehabilitation are the main advantages compared with the closing wedge technique [10].

The success of MOWHTO is mainly dependent on several factors, such as good selection of patients, adequate preoperative planning, method of fixation, and intactness of the lateral hinge [11]. The risk of complications varies from 37 to 55% in the literature, including lateral hinge fractures, infection, compromised wound healing, DVT, nonunion, limb length mismatch, under or over correction, metalwork irritation, and implant failure [12–14].

Maintenance of the lateral hinge during surgery is a key factor for successful postoperative outcomes, stability of the osteotomy site, and early bone healing [15]. A fracture of the lateral hinge results in loss of correction, delayed bone healing, and prolonged recovery [16]. Takeuchi et al. [17] recorded an incidence of 25% for lateral hinge fractures (LHF) during surgery and described three types of fractures according to their location. Type I occurs at the level of the tibiofibular joint; Type II is located below the tibiofibular joint; and Type III extends to the lateral tibial plateau.

Some clinicians suggested several solutions to minimize the risk of intraoperative lateral hinge fractures and prevent their problematic consequences, like anteroposterior drilling of the hinge apex, fluoroscopic control of the cut depth, and using patient-specific cutting guides [2, 18, 19]. One of the proposed solutions is the application of two protective K-wires crossing 1 cm before the lateral cortex [20].

This study describes a method of K-wire insertion from lateral to medial to facilitate the surgical technique by clearing the medial working side. The goal of this study is to assess the functional outcomes and the role of using additional protective lateral K-wires during MOWHTO to minimize the risk of intraoperative lateral hinge fracture. It was hypothesized that the application of lateral crossing K-wires during the procedure will reduce the risk of intraoperative iatrogenic lateral hinge fractures.

## Patients and methods

The data of 48 patients who underwent MOWHTO between May 2021 and August 2022 at our institution were collected retrospectively from their medical files after obtaining approval from our institutional research board (IRB) unit. The patients signed informed consent before surgery, and the study was carried out in line with the principles of the Declaration of Helsinki for human studies. The patients were evaluated according to treatment method; one group had MOWHTO using additional lateral protecting K-wires (+ MOWHTO), while the other group did not use protecting K-wires (-MOWHTO). The included patients were operated on based on surgeon preference to investigate the efficacy and clinical relevance of the protecting lateral wires.

Patients complaining of medial joint pain due to varus knee deformity with MTPA less than 85^°^, Albach grade 1, 2 medial compartment osteoarthritis, and age less than 60 years were eligible for inclusion in this study. Patients with Albach grade 3 or more medial osteoarthritis, associated patellofemoral or lateral compartment arthritis, post-traumatic osteoarthritis, rheumatoid arthritis, patients who had concomitant ligamentous, or chondral surgeries, and incomplete medical records were excluded from the study.

The demographic information of the treatment groups is listed in Table 1.

**Table 1:** Patient criteria in the treatment groups

	+MOWHTO group (*N* = 24)	-MOWHTO group (*N* = 24)	*P* value
**Age (mean ± SD**,** range) (years)**	41.8 ± 3.7 (36–49)	43.5 ± 4.06 (38–50)	0.12
** Sex**			
Male Female	15 (62.5%) 9 (37.5%)	17 (70.8%) 7 (29.2%)	0.76
**Occupation**	9 housewives 3 plumbers 4 carpenters 3 factory workers 1 teacher 4 employees	7 housewives 1 painter 2 builders 2 laborers 5 factory workers 1 lawyer 6 employees	
**The planned angle of correction** **(degree)**	10.1 ± 2.7	10 ± 2.5	0.91
**Method of fixation**			
Tomofix plate Proximal medial tibial plate Pudu plate	23 1 -	21 1 2	0.61
**Autograft usage (n)**	12/24	10/24	0.77
**Follow-up period (months)**	30.5 ± 3.6 (25–37)	31.6 ± 3.2 (27–36)	0.26

Data are expressed as numbers (n), percentages (%), and means ± SD_s_ (range)

### Preoperative evaluation

**Fig. 1:**
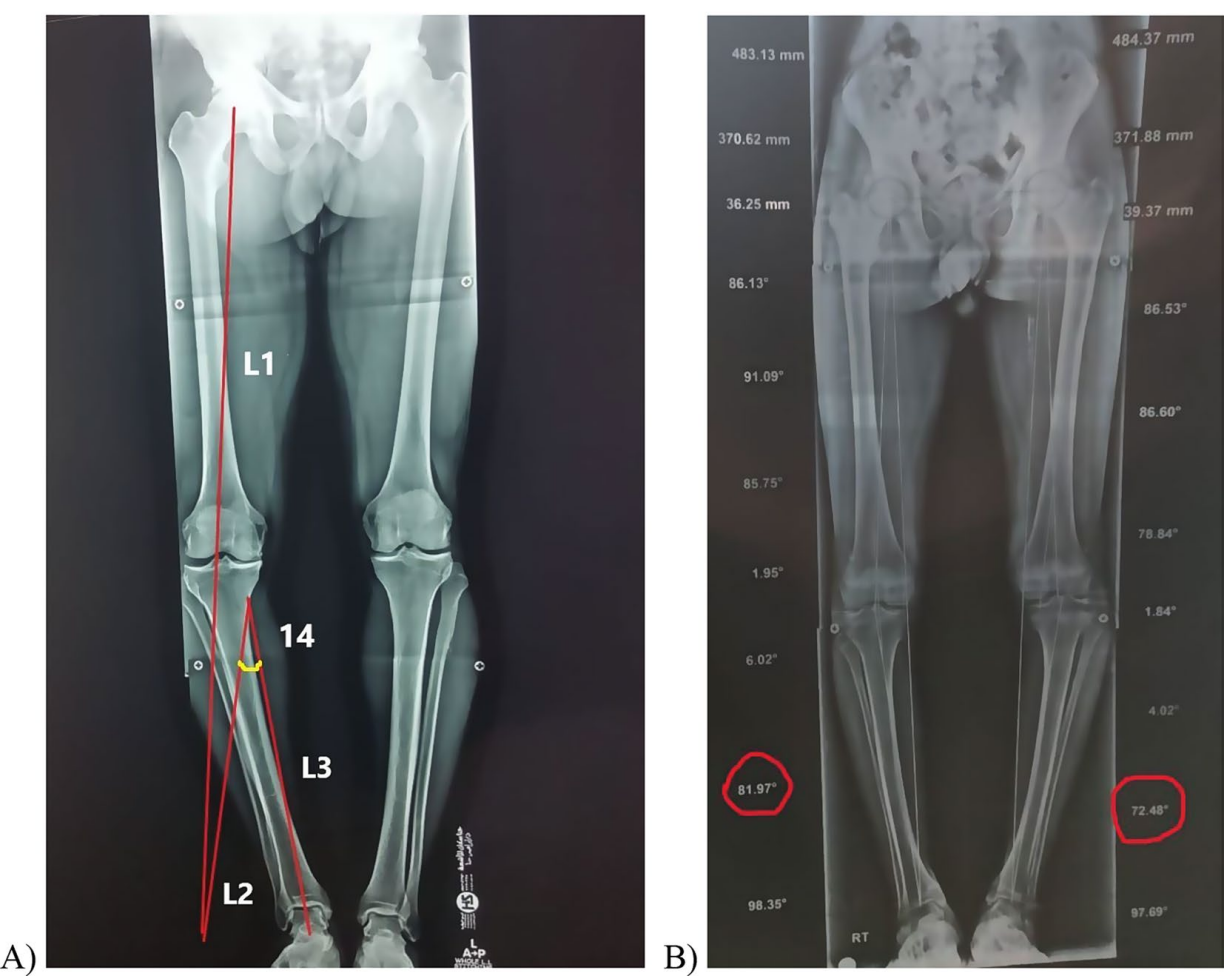
(**A**) The correction angle is marked on a long X-ray film using the Miniaci method. It measures 14^°^ and lies between L2 and L3. L1 starts from the hip center to an imaginary point of the anticipated ankle position following correction, L3 passes from the center of the ankle to the starting point of the osteotomy at the medial cortex, and L2 connects L1 and L3. (**B**) The recorded MPTA is marked with a red circle. MPTA = medial proximal tibial angle

The patients were examined clinically for limb alignment, meniscal injuries, ligamentous insufficiencies, patella-femoral, and lateral compartment osteoarthritis. Standing anteroposterior and lateral radiographs were used to assess the degree of medial arthritis using the Albach grading system [21] and the involvement of other compartments. The MTPA and angle of correction were estimated on long weight-bearing digital X-ray films including hips, knees, and ankles using PaxeraHealth software (Boston, USA) by a single radiologist. The angle of correction was determined using the Miniaci method [22] (Fig. 1). MRI was used for the detection of other intra-articular lesions. The knee injury and osteoarthritis score − 12 (KOOS-12) [23] was used for functional assessment.

### Surgical technique

**Fig. 2:**
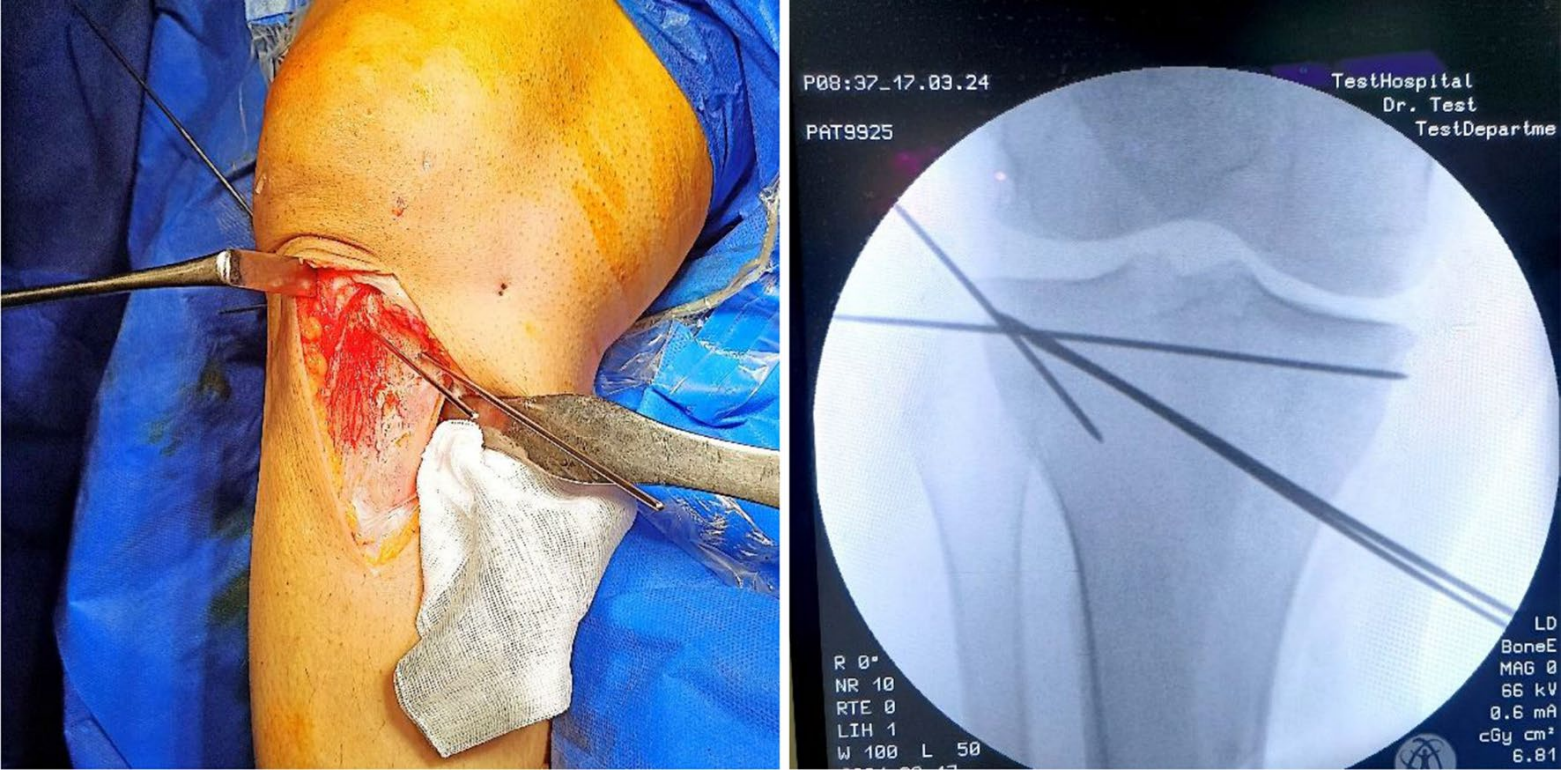
Two parallel medial K-wires are drilled as a guide for the osteotome, and additional lateral wires are inserted for lateral hinge protection

**Fig. 3:**
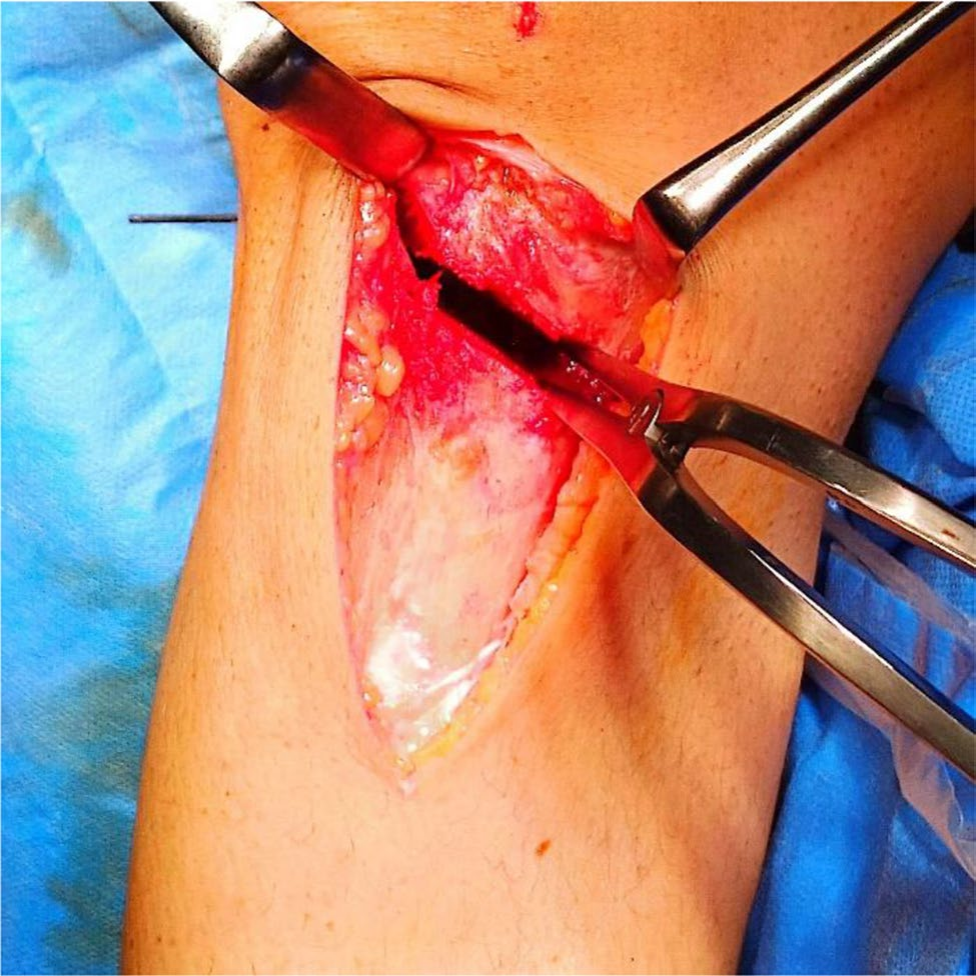
A laminar spreader is placed posterior for gradual distraction of osteotomy

All surgeries were performed under spinal anaesthesia by two different surgeons. A prophylactic antibiotic of 1 g intravenous cefazoline was administered 30 min preoperatively. The patient was placed supine on the operating table, and a well-padded tourniquet was applied to the affected limb. A routine diagnostic knee arthroscopy was the first step of the procedure to check for the integrity of intraarticular structures. A longitudinal skin incision started proximally from the posteromedial aspect of the tibial plateau, reaching distal to the tibial tuberosity anteriorly. A subperiosteal release of the superficial medial collateral and pes anserinus was done with a sharp scalpel, and the Hohmann retractors were placed behind the patellar tendon and posterior surface of the upper tibia for protection. Two parallel K-wires were drilled 3 to 4 cm below the medial joint line, aiming laterally to the proximal part of the fibular head, 1.5 cm below the lateral joint line, and 10 mm before the lateral cortex under fluoroscopic guidance. Two additional 1.8 mm K-wires were inserted from lateral to medial; one passed parallel to the tibial plateau to avoid intraarticular fracture propagation, and the other was drilled parallel to the lateral cortex from proximal to distal, crossing with the superior wire 1 cm medial to the lateral cortex for protection of the lateral hinge (Fig. 2). The bone was osteotomized using a power saw parallel to the medial guiding wires, stopping at the crossing point of the lateral wires. The posterior cortex at the osteotomy site was drilled with a 3.2 mm drill bit and cut using a sharp osteotome. The osteotomy was gradually opened using a laminar spreader placed posterior to maintain the normal tibial slope (Fig. 3). The desired correction is confirmed on fluoroscopic imaging using the cable of the diathermy device from the hip center to the center of the ankle, passing through a point just lateral to the lateral tibial spine. An autogenous tricortical iliac graft was inserted for gaps greater than 10 mm, and the osteotomy site is secured with plate and screws. Finally, the wound was closed in layers over a suction drain.

### Postoperative rehabilitation and follow-up

**Fig. 4:**
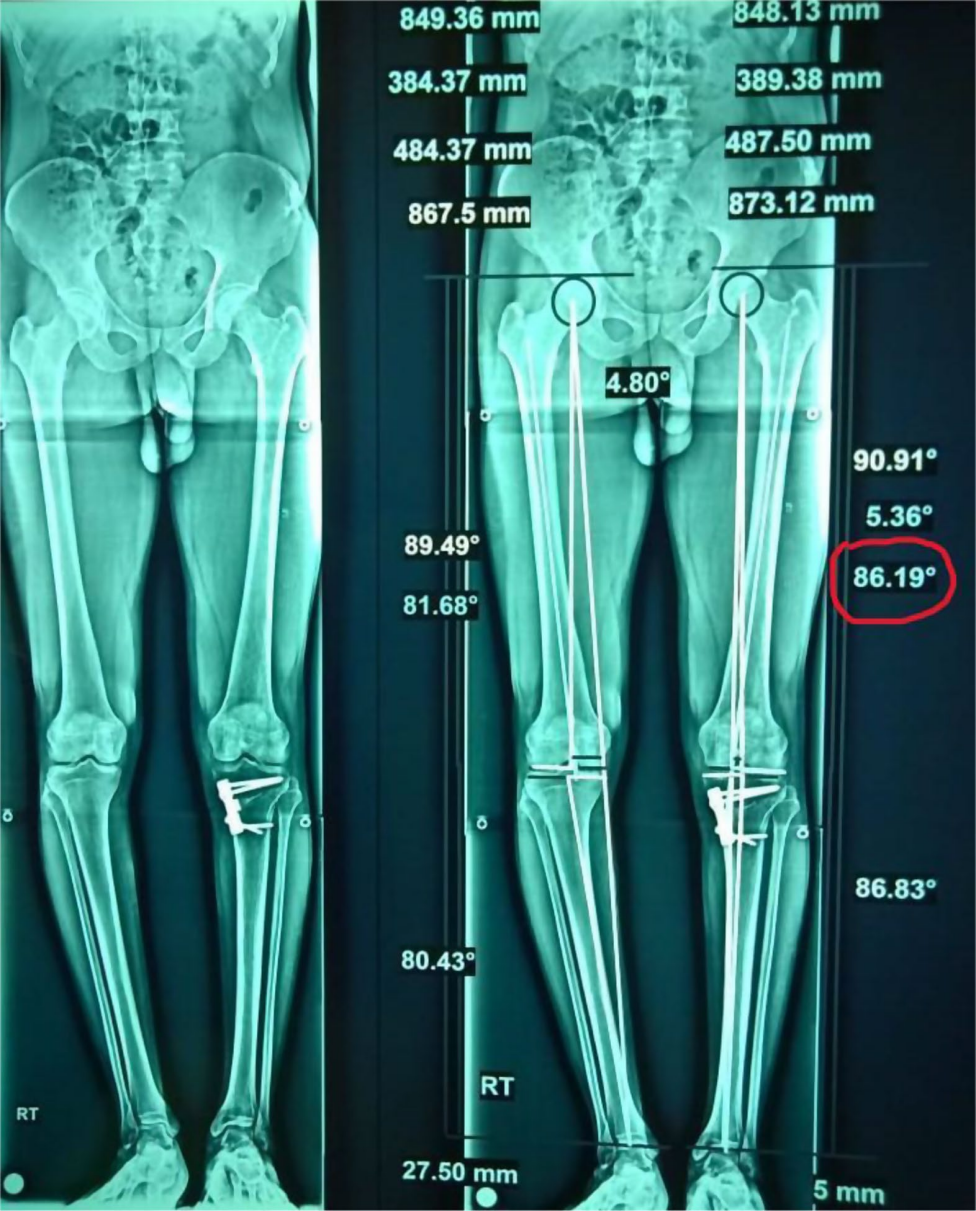
The final postoperative MPTA is calculated on a long X-ray radiograph (red circle)

The antibiotic continued for 24 h postoperatively. The drain was retained for two days, and stitches were removed 14 days after surgery. Knee motion started early, and partial weight bearing was allowed. Full weight bearing was achieved at 6 weeks postoperative if there were no hinge fractures. For type 1 fractures, partial weight bearing was recommended for 6 weeks, then progressed to full weight bearing. Weight-bearing was delayed for 6 weeks for patients who had type 3 fractures; partial weight bearing for the next 6 weeks; and full weight bearing was encouraged at 12 weeks postoperative. Bone healing was evaluated on serial radiographs at 4, 8, 12, 16, 20, and 24 weeks postoperative, and the MPTA was recorded on the final follow-up long X-ray films (Fig. 4). The KOOS-12 was also reported for functional assessment at 24 months postoperative.

### Statistical analysis

The IBM SPSS Statistics for Windows, Version 23.0 (Armonk, NY: IBM Corp. 2015) was used for statistical analysis. The Shapiro test justified the normality of data, and quantitative data were presented as mean ± standard deviation (SD). The independent t-test and paired t-test were employed to compare two groups of normally distributed numerical variables, while the categorical variables were compared using the Fisher Extract test. The odds ratio was used to estimate the probability of lateral hinge fractures in each group. All statistical tests were two-sided, with a p-value < 0.05 deemed statistically significant. A post hoc analysis of the statistical test power was 90.8% for the given sample size and α error of 0.05 using the G*Power software calculator (version 3.1).

## Results

Among 63 patients who underwent MOWHTO between May 2021 and August 2022, only 48 patients met the eligibility criteria for inclusion in this study. Four patients who had concomitant focal chondral lesions, eight cases with anterior cruciate ligament injuries, and three cases with deficient medical records were excluded. Both treatment groups did not show significant differences regarding mean age, sex, planned angle of correction, usage of bone graft, and follow-up time (Table 1).

**Fig. 5:**
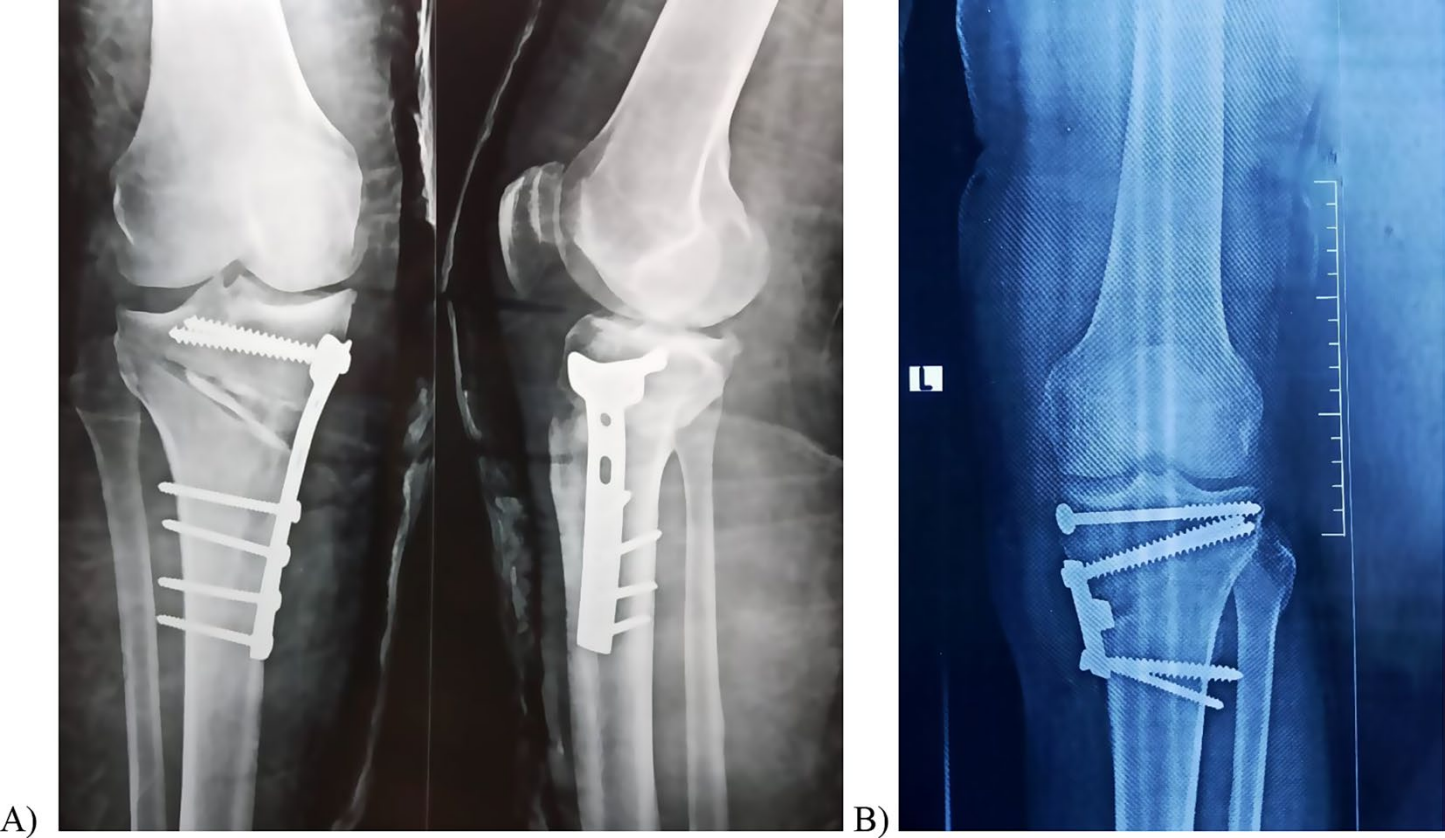
Radiographs show type I (**A**) and type III (**B**) lateral hinge fractures fixed with an extra 6.5 mm cancellous screw

The mean operative time was slightly prolonged in the + MOWHTO group, with no statistical difference between both groups (58.2 ± 7.4 vs. 56.5 ± 4.3) (*p* = 0.34) (Table 2). There was no significant difference displayed between both groups regarding methods of fixation (Table 1). One patient had type I iatrogenic intraoperative lateral hinge fracture in the + MOWHTO group, while there were five type I fractures and two type III fractures in the -MOWHTO group under image intensifier (odds ratio 0.11 vs. 9.4, respectively) (*p* = 0.04) (Fig. 5). The MPTA and KOOS-12 showed highly statistical differences before and after surgery (p ˂ 0.00001) in both treatment groups (Table 2); however, the final postoperative results did not have significant differences between groups (Table 2). The + MOWHTO group had statistically significant shorter time of union and return to work (*P* = 0.001 and 0.002, respectively) (Table 2). A smaller complication rate was observed in the + MOWHTO group with no statistical difference between both techniques (*P* = 0.66). Concerning the recorded morbidities in the + MOWHTO group, one patient (4.2%) had a superficial wound infection and was treated with repeated wound dressing, and one patient (4.2%) developed a limited range of motion in the last 30^°^ of knee flexion. In the -MOWHTO group, two patients (8.4%) had bone superficial wound infection; one had wound dehiscence that healed using a vacuum-assisted closure (VAC) device; and one developed DVT in the calf that was treated with anticoagulants for 6 months until the recanalization of the thrombosed vein.

**Table 2:** Final clinical and radiological outcomes

	+MOWHTO group (*N* = 24)	-MOWHTO group (*N* = 24)	*P* value
**Operative time (minutes)**	58.2 ± 7.4	56.5 ± 4.3	0.34
**Time of union (weeks)**	12.38 ± 2.2	15.17 ± 3.4	0.001*
**Return to work (weeks)**	16.3 ± 2.4	19.1 ± 3.3	0.002*
**Type of LHF**			
Type I Type II Type III	1 (4.2%) - -	5 (20.8%) - 2 (8.3%)	0.04*
**KOOS-12**			
Preoperative Postoperative P value	58.2±7.1 94.6±5.03 0.00001	59.2 ± 9.6 92.4 ± 6.9 0.00001	0.68 0.21
**MPTA (degree)**			
Preoperative Postoperative P value	80.7 ± 1.8 90.8 ± 2.3 0.00001	79.5 ± 2.1 89.5 ± 2.3 0.00001	0.051 0.07

The data were presented as means ± SD. LHF = lateral hinge fractures; KOOS-12 = knee injury and osteoarthritis outcome score; MPTA = medial proximal tibial angle. P-value ˂ 0.05 is rated statistically significant

## Discussion

This study compared the results between two groups of patients; one group adds lateral crossing K-wires for the protection of the lateral hinge during OWHTO and the other did not. The key results are that the group using lateral protecting K-wires during MOWHTO had a lower risk of lateral hinge fractures than the group that did not, with statistically significant shorter bone healing times, and an earlier return to work. The hypothesis has been validated and has contributed to the limited number of clinical and biomechanical trials that are currently available.

The incidence of iatrogenic lateral hinge fractures during OWHTO ranges from 3 to 30%, with 18% occurring intraoperatively [24]. The role of an intact lateral cortical hinge for maintenance of postoperative angular correction had been justified in previous studies [25, 26]. A recent biomechanical study by Kang et al. [27] revealed that Takeuchi type I fractures had the same stability as an intact hinge, while type II and III fractures were unstable, which may be associated with a delayed union and under correction. Our study reported an overall lateral hinge fracture incidence of 16.6%, with 4.2% being unstable (type III) in the -MOWHTO group.

However, several prognostic parameters, such as age, body mass index, width of tibial plateau, and site of osteotomy, were investigated for their relationship with the frequency of lateral hinge fractures, the dimension of the opening gap was the most significant correlation throughout the literature [28]. Aryee et al. [29] suggested the usage of bone grafts for medial gaps of more than 1 cm. As a result, twelve patients (50%) in the + MOWHTO group and ten patients (41.7%) in the -MOWHTO group received autogenous tricortical iliac bone grafts for osteotomy gaps larger than 10 mm, with no statistical difference between groups (*p* = 0.77).

Our study used the lateral side for the application of protecting wires, contrary to Gulagaci et al. [30]. The possible advantage of lateral to medial K-wire insertion is that it avoids interference with the working medial side, which may be cumbersome for the surgeon during surgery. The application of crossing lateral wires may theoretically increase the mean surgical time in the + MOWHTO group, however we did not find significant difference between both groups (Table 2). This may be attributable to the lower rate of intraoperative hinge fractures, which may extend the time required for precise correction and further fixation. Gulagaci et al. [30] did not discover a difference regarding the time of surgery between treatment groups. This point will need further prospective investigation on a large scale of patients in the future.

Several strategies have been developed to lessen the possibility of lateral hinge fractures during OWHTO, with no consensus on any of them [31]. The recently described protective K-wires technique produced encouraging results that were supported by clinical and biomechanical research [20, 30]. The rationale of this method is to control the depth of the osteotomy cut, dissipate stresses, and provide sufficient mechanical resistance of the lateral hinge to breakage [20]. A recent biomechanical investigation by Ozmen et al. [32] demonstrated that protecting wires with diameters smaller than 2.5 mm was insufficient to avoid bone breaks in finite element analysis. We utilized 1.8 mm K-wires during surgery. This research displayed a lower risk of lateral hinge fractures in the + MOWHTO group compared to the other group (OR 0.13 vs. 7.6). Our findings were consistent with those presented by Gulagaci et al. [30].

Delayed bone healing and non-union are some of the anticipated implications of lateral hinge fractures [33]. Dorofeev et al. [34] found an elevated rate of non-union in patients with lateral hinge breaks. The rate of revision surgeries was 10.3% in their series. Song et al. [35] observed a considerably shorter time of union in patients with intact lateral hinges relative to the fracture group (*P* < 0.001). Our series found statistically significant earlier time to union and return to work in the + MOWHTO group in comparison to the – MOWHTO owing to the lower rate of LHF. Similar results were documented by Gulagaci et al. [30]. Kim et al. [36] noticed that patients with type II and III lateral hinge fractures had delayed return to their activities. Duivenvoorden et al. [37] reported that quicker return to work is an important metric of success for OWHTO, especially in younger, active patients, reinforcing the clinical relevance of this finding.

Loss of correction is another adverse effect related to intraoperative lateral hinge failure because of the difficulty in maintaining the desired position during fixation of the osteotomy even in stable type I fractures [38]. Even though we observed slightly better postoperative MPTA in the + MOWHTO group, the variability was not significant among the studied groups (*p* = 0.07). Also, Gulagaci et al. [30] discovered similar findings in their series. Kim et al. [36] did not find significant alterations in the postoperative radiographic alignment among patients with type I and II fractures owing to fixation with angular-stable plating systems. Tomofix plates were used for fixation in 91.6% of patients in our series.

The functional KOOS-12 in the + MOWHTO did not show significant variance compared to the other group at 24 months follow-up. These results were in following the findings of Gulagaci et al. [30] in their study. Kim et al. [36] concluded that type I and II LHF did not negatively alter the final KOOS records.

The total postoperative complication rate was 12.5% in both groups, with no statistically significant difference between them (*p* = 0.66). The + MOWHTO group recorded one patient who suffered from superficial wound sepsis, and one patient had a loss of the last 30^°^ of flexion, while the other group had two cases of superficial surgical site contamination, one case of wound dehiscence, and one patient with DVT. Our results were near to Gulagaci et al. [30] who reported a complication rate of 15.8% with no remarkable variation between treatment groups.

### Limitations of the study

This study had some limitations that should be mentioned. First, the data were analyzed retrospectively without randomization, which may lead to selection bias. The number of patients is small, but it was sufficient to produce 90% power for statistical tests in the post hoc analysis. A larger sample will be recommended for generalization of the results. Although it is a short-term study, there is no need for a longer follow-up period as the lateral hinge fractures are intraoperative events or discovered immediately postoperative. The diagnosis of LHF was based mainly on intraoperative fluoroscopy and postoperative radiographs, as some patients could not afford the cost of postoperative CT scan. The procedures were done by two different surgeons, which may disrupt the homogeneity of outcomes. Finally, there is no comparison with other preventive techniques, like patient-specific guide devices or apical drilling, and more clinical and biomechanical investigations will be beneficial to prove the efficacy of this method in the future.

## Conclusion

The results of this study proved the efficacy of using lateral crossing K-wires for protection against lateral hinge fractures during MOWHTO. The + MOWHTO group experienced a lowered risk of intraoperative iatrogenic fractures with no relevant difference in the operative time between both techniques. The time to bone union and resume the previous daily activities were faster in the + MOWHTO group, whereas the final functional KOOS-12 score and postoperative MPTA did not exhibit a significant difference in respect to the other group.

## Data Availability

No datasets were generated or analysed during the current study.

## Abbreviations

MOWHTO: Medial open wedge high tibial osteotomy
MPTA: Medial proximal tibial angle
KOOS: Knee injury and osteoarthritis outcome score
mm: millimeters
SD: standard deviation
SPSS: Statistical Package for Social Sciences
HTO: High tibial osteotomy
LHF: Lateral hinge fracture
DVT: Deep venous thrombosis
g: gram
MRI: Magnetic resonant imaging
VAC: Vacuum assisted closure
